# MicroRNAs in Valvular Heart Diseases: Potential Role as Markers and Actors of Valvular and Cardiac Remodeling

**DOI:** 10.3390/ijms17071120

**Published:** 2016-07-13

**Authors:** Cécile Oury, Laurence Servais, Nassim Bouznad, Alexandre Hego, Alain Nchimi, Patrizio Lancellotti

**Affiliations:** 1 GIGA-Cardiovascular Sciences, Interdisciplinary Cluster for Applied Genoproteomics (GIGA), University of Liège, 4000 Liège, Belgium; laurence.servais@doct.ulg.ac.be (L.S.); Nassim.Bouznad@med.uni-muenchen.de (N.B.); alexandre.hego@hotmail.fr (A.H.); alainnchimi@gmail.com (A.N.); plancellotti@chu.ulg.ac.be (P.L.); 2 Department of Cardiology, University of Liège Hospital, 4000 Liège, Belgium; 3 Experimental and Molecular Pathology Laboratory, Insitute of Pathology Ludwig Maximilians, University Munich, 80331 Munich, Germany; 4 Gruppo Villa Maria Care and Research, Anthea Hospital, 70124 Bari, Italy

**Keywords:** microRNAs, valvular heart diseases, biomarkers, cellular and animal models

## Abstract

miRNAs are a class of over 5000 noncoding RNAs that regulate more than half of the protein-encoding genes by provoking their degradation or preventing their translation. miRNAs are key regulators of complex biological processes underlying several cardiovascular disorders, including left ventricular hypertrophy, ischemic heart disease, heart failure, hypertension and arrhythmias. Moreover, circulating miRNAs herald promise as biomarkers in acute myocardial infarction and heart failure. In this context, this review gives an overview of studies that suggest that miRNAs could also play a role in valvular heart diseases. This area of research is still at its infancy, and further investigations in large patient cohorts and cellular or animal models are needed to provide strong data. Most studies focused on aortic stenosis, one of the most common valvular diseases in developed countries. Profiling and functional analyses indicate that miRNAs could contribute to activation of aortic valve interstitial cells to a myofibroblast phenotype, leading to valvular fibrosis and calcification, and to pressure overload-induced myocardial remodeling and hypertrophy. Data also indicate that specific miRNA signatures, in combination with clinical and functional imaging parameters, could represent useful biomarkers of disease progression or recovery after aortic valve replacement.

## 1. Introduction

Valvular heart disease (VHD) is a major leading cause of cardiovascular mortality. According to the most recent statistics, the prevalence of any VHD in the entire U.S. population is 2.7%, with 0.4% aortic stenosis, 0.5% aortic regurgitation, 0.1% mitral stenosis, and 1.7% mitral regurgitation [1]. Prevalence of VHD increases with age, reaching about 13% in patients ≥75 years old [2].

## 2. Valvular Heart Disease Pathophysiology

### 2.1. Aortic Stenosis

The prevalence of moderate or severe aortic stenosis (AS) in patients ≥75 years equals 2.8% [2]. No medical therapy currently exists for AS, and approximately 50% of patients with severe AS are admitted for cardiothoracic surgery, and about 40% undergo aortic valve replacement. Calcific aortic stenosis (AS) is one of the most common VHD in developed countries. In addition to older age, congenital anomalies of the aortic valve (bicuspid valve) are major risk factors for calcific AS.

The pathophysiological mechanisms of calcific AS are complex and remain poorly understood. Lipoprotein deposition, chronic inflammation, osteoblastic transition of valve interstitial cells and active leaflet calcifications are likely to be involved.

A hallmark of disease progression is a misalignment of collagen fibres within the fibrosa layer, proximal to the aorta that produces thickened, fibrotic leaflets, and overall stiffened leaflet with altered biomechanical functionality. Later accumulation of calcific nodules worsens the phenomenon. Alterations of leaflet dynamics during cardiac cycle results in modified shear stresses, leading to phenotypic changes of leaflet lining valve endothelial cells (VEC), and infiltration of inflammatory cells in the valve tissue. In addition, VEC can undergo mechanical strain and Wnt/β-catenin signaling-dependent endothelial-to-mesenchymal transition, invade the leaflet interstitium, and play an active role in valve remodelling. These phenotypic changes mainly occur on the fibrosa side of the valve leaflets, subjected to more disturbed flow than the ventricular side. Differentiation of valve interstitial cells (VIC) in myofibroblasts (expressing smooth muscle cell protein markers) would be responsible for fibrotic accumulation of collagen during the initial stages of calcific AS. Osteoblastic differentiation of VIC would lead to the formation of the osteogenic nodules in valve leaflets. Whether the fibrotic and calcific processes are interrelated is uncertain. Mechanistically, active transforming growth factor (TGF)-β1 controls the expression of proteins associated with VIC myofibroblast transition [3]. In vitro incubation of VICs with TGF-β1 induces the expression of aortic smooth muscle actin and production of type I collagen. TGF-β1 also causes the formation of apoptosis-driven dystrophic calcific nodules that occurs as a response to augmented contractility and intercellular tension. Activation of the Wnt/β-catenin signalling pathway promotes myofibroblastic activation through non-canonical TGF-β1-dependent activation of p38 mitogen-activated protein kinase (MAPK). In addition to TGF-β1, elevated levels of Bone morphogenetic protein 2 (BMP-2) and BMP-4 expression, two pro-osteogenic cytokines belonging to the TGF-β superfamily, have been observed in stenotic calcific aortic valve disease (CAVD) leaflets.

Chronic inflammation is a major feature of calcific AS. Inflammatory processes are associated with, and may be responsible for the alterations in the valve extracellular matrix (ECM) through release of proteases by inflammatory cells. Infiltrating macrophages may also actively contribute the deposition of calcium phosphate minerals.

Recently, it has been proposed that lipid accumulation in the valve leaflet would promote the inflammatory response and lesion development in early calcific AS, leading to fibrocalcific remodelling. Lipoprotein(a), composed of a low density lipoprotein (LDL)-like particle in which apolipoprotein(a) is covalently bound to apolipoprotein B, and VIC-derived autotaxin, a member of the ectophosphodiesterase/nucleotide phosphohydrolase (ENPP) family converting lysophosphatidylcholine to lysophosphatidic acid, may induce valve calcification by regulating inflammation-induced bone morphogenetic protein (BMP) [4]. Expression of NPP1 and 5′-nucleotidase by VICs could mediate mineralization of the aortic valve through the adenosine A2 receptor [5]. Furthermore, shear stress encountered in stenotic aortic valve activates platelets, generating platelet microparticles, and cause profound hemostasis disorders mainly due to the loss of high molecular weight von Willebrand factor multimers [6,7]. Tissue factor, the key initiator of coagulation expressed within the valve itself, is likely to be involved in fibrin deposition, infiltration of lipid and macrophages. Thus, prothrombotic and inflammatory mechanisms likely promote the progression of disease.

In addition to the valve alterations, the AS-associated chronic hemodynamic stress causes left ventricle remodelling due to cardiomyocyte hypertrophy and increased production of ECM proteins in the myocardium. The resulting contractile alteration can evolve to overt heart failure. TGF-β is a key player in pathological remodeling of the heart that occurs in response to pressure overload through the induction of interstitial fibrosis and cardiomyocyte hypertrophic growth [8].

In AS, aortic valve replacement (AVR), either surgical or via transcatheter implantation, is the only treatment that improves survival [9]. A major management challenge is deciding on the correct timing of aortic valve replacement. In this context, biomarkers could be very useful for the establishment of risk stratification in asymptomatic patients and the identification of determinants of poor prognosis in symptomatic subjects. A key element would be to identify patients with asymptomatic severe AS who may benefit from early AVR. AVR is indicated in patients with severe AS who develop symptoms and/or left ventricular (LV) systolic dysfunction (LV ejection fraction < 50%). At earlier stages of the disease, LV ejection fraction often remains normal even though the alterations in LV myocardial structure and function can already be irreversible. Such LV remodelling process may occur in patients with moderate AS, in the absence of clear symptoms, and can trigger biomarkers release (i.e., B-type natriuretic peptide (BNP)).

### 2.2. Mitral Valve Disorders

Mitral valve disease is the other most common valvular lesion, and a frequent cause of heart failure and mortality. Moderate mitral regurgitation occurs at a frequency of 9.3% in patients aged ≥75 years [2]. According to Carpentier’s functional classification [10], the frequency order is as follows: Type I (congenital mitral regurgitation and endocarditis) < Type IIIa (rheumatic heart disease, systemic lupus erythematosus, antiphospholipid syndrome) < Type II (myxomatous mitral regurgitation) < Type IIIb (ischemic mitral regurgitation, LV dysfunction, dilated cardiomyopathy).

Mitral valve is composed of the annulus, anterior and posterior leaflets, and chordae that attach the leaflets to papillary muscles. The normal mitral leaflets comprise three tissue layers (atrialis, spongiosa, and fibrosa/ventricularis) and a continuous endothelial surface layer. VICs are located in the deep sub-endothelial layers. The fibrosa contains collagen fibres that are aligned parallel to the leaflet free edge. This layer is connected to the mitral annulus and faces the LV.

Structural alterations in the mitral valve and secondary changes due to ventricle remodelling both contribute to the development of valve insufficiency. Mitral valve insufficiency affects cardiac function mechanically, because of required elevated filling pressure, but also results in impaired contractility and electrical instability.

Mitral regurgitation (MR) represents a leakage of blood from the left ventricle backwards into the left atrium during systole, which may be due to a primary abnormality (often referred to as organic MR) of one or more components of the valve apparatus or may be secondary (often referred to as functional MR) to LV dysfunction.

The main causes of primary MR include degenerative mitral valve diseases, such as myxomatous degeneration leading to eventual mitral valve prolapse, fibroelastic deficiency disease, acute rheumatic carditis, infective endocarditis, congenital diseases, use of certain drugs, and mitral annulus calcification. Secondary causes of MR include coronary artery disease (CAD), dilated cardiomyopathy, and hypertrophic cardiomyopathy.

Mitral valve prolapse can be a feature of myxomatous valve disease, but can also occur in patients with normal mitral valve leaflets. Besides, prolapse and mitral valve leaflet thickening can be observed in several inherited connective tissue disorders, including Marfan syndrome, and Ehlers-Danlos syndrome.

In ischemic mitral regurgitation (IMR), which often affects patients surviving a myocardial infarction or with dilated cardiomyopathy, leaflet area cannot compensate LV dilatation, and the leaflets become stiffer and undergo fibrosis. Remodelling or distortion of LV structure leads to papillary muscle displacement, leaflet tethering, and impaired coaptation, with a substantial risk of death [11]. In the ischemic setting, it is of paradigm importance to improve the understanding of the adverse cellular and mechanical processes leading to mitral leaflet insufficiency, and regurgitation.

Ischemic MR reflects maladaptive valve apposition relative to changes in LV geometry. Using animal models of ischemic MR, investigators have reported that leaflet thickening results from a dynamic adaptation to increased leaflet stresses. Reactivation of endothelial-to-mesenchymal transition has been described with possible roles for TGF-β, BMP, vascular endothelial growth factor, and Notch signalling.

Upregulation of TGF-β1 has been reported in animals and humans with MVP and is thought to contribute to extracellular matrix remodeling and fibrosis [12]. A recent mRNA profiling study [13] indicates that (1) TGF-β signaling is increased in myxomatous mitral valve tissue because of increased ligand expression and derepression of canonical mothers against decapentaplegic homolog (SMAD) 2/3 signaling; (2) canonical BMP and Wnt/β-catenin signaling pathways are increased in myxomatous mitral valve degeneration (MMVD); (3) TGF-β, BMP, and Wnt/β-catenin pathways in MMVD are associated with matrix remodeling, procalcific and proproliferative cellular processes; and (4) activated immune cells are localized to myxomatous mitral valves, which possibly contribute to maladaptive wound repair response. Fibrogenic, osteogenic, and proliferative signaling are robustly activated in myxomatous valve tissue, and derepression of these signaling cascades may be a key permissive mechanism promoting disease progression. Although some of these changes appear reminiscent of the molecular signature present in calcific aortic valve disease, it is critical to note that the phenotypic consequences of these changes are dramatically different and are likely to act in a highly context-dependent manner.

Thus, mitral valve is a dynamic structure that adapts to continuous mechanical and biological stresses and is influenced by ventricular pathology. Such valve plasticity depends on several factors, including signals intrinsic to the valve, paracrine signals from the left ventricular wall, activation of the endothelium, and possible engraftment of blood-borne cells.

Surgery, mitral valve replacement or repair, is the only therapy available for severe symptomatic primary MR, especially in organic forms of MR [14].

## 3. miRNA Expression in Valvular Heart Disease (VHD): Profiling Studies

In both aortic and mitral valve diseases, early detection and modification of the mechanisms of disease progression could limit valve degeneration and related clinical complications [15]. Over the time, symptoms can develop or ejection fraction can deteriorate and lead to irreversible LV impairment. Biomarkers that reflect the degree of severity of valve disease and early LV dysfunction would be highly valuable. In the last decade, a big focus has been made on natriuretic peptides (BNPs). BNPs, produced in response to myocardial wall stress, correlate well with disease severity and symptomatic status and can also be useful for prognosis [16,17,18]. BNPs have a role in the risk stratification of VHD patients as well as in routine surveillance and monitoring, and are being incorporated into clinical management together with functional imaging modalities [19]. However, additional biomarkers are still required to identify early stages of disease progression in asymptomatic patients, and/or to better define correct timing of surgery to preserve ventricular function.

MicroRNAs (miRNAs) might fulfill this role. miRNAs are small non coding RNA of about 21-nucleotide (nt)-long that regulate gene expression at the post-transcriptional level [20]. More than 1000 miRNA have been identified in humans, possibly targeting ~30% of human genes [21]. Target mRNAs and biological roles have been assigned to only a few dozen miRNAs although almost every process investigated seem to be regulated by miRNAs. The expression of many miRNAs shows tissue- or developmental stage-specific patterns. Most importantly, miRNA signatures have been associated with a wide panel of human diseases [22]. In the cardiovascular field, circulating miRNAs are currently investigated as biomarkers in acute myocardial infarction and heart failure [23]. In addition, miRNAs likely represent key actors of biological processes underlying cardiovascular disorders, such as LV hypertrophy, ischaemic heart disease, heart failure, hypertension and arrhythmias. In this context, the present review aims at giving an overview on current knowledge of the role of miRNAs in the most common VHDs.

### 3.1. Aortic Stenosis

#### 3.1.1. Aortic Valves

Five studies investigated the expression of miRNAs in aortic valve leaflets from AS patients undergoing AVR, by using a miRNA candidate approach or miRNA microarray (Table 1). Microarray data were validated by quantitave real-time polymerase chain reaction (qPCR) unless indicated. The first study by Nigam et al. [24] analyzed bicuspid aortic valve samples from patients with AS or aortic insufficiency (AI), indicating a downregulation of miR-26a, miR-30b, and miR-195 in the aortic valves of patients requiring AVR due to AS, compared to AI. Yanagawa et al. [25] compared miRNA microarray profiles in bicuspid valves vs. tricuspid valves of AS patients. 35 miRNA were differentially expressed between bicuspid and tricuspid valves. Downregulation of miR-141 was confirmed by qPCR. Zhang et al. [26] found a downregulation of miR-30b (qPCR) in calcific tissue of aortic valves vs. non-calcific adjacent tissue. In the study by Patel et al. [27] Bicuspid aortic valves BAVs expressed lower levels of miR-148-3p as compared to control aortic valve leaflets. Ohukainen et al. [28] performed a microarray analysis of calcific stenotic aortic valves and control valves and identified 3 downregulated miRNAs (miR-374b*, miR-602, miR-939) and upregulation of miR-125b.

#### 3.1.2. Left Ventricle

Five other studies aimed at identifying differential miRNA expression in LV biopsies from AS patients requiring AVR vs. surgical controls or from AS patients with severe or non-severe fibrosis. Two of them indicate that miR-133a is not only upregulated in myocardium of AS patients, but also carries predictive value of LV mass normalisation one year after valve replacement [29,30]. Villar et al. [31] found elevated levels of miR-21 in AS patients compared to controls. Patients with left ventricular hypertrophy due to AS showed reduced levels of miR-1, which was restored after transcatheter aortic valve implantation [32]. By miRNA microarray analysis, Beaumont et al. [33] found 99 downregulated miRNAs and 19 up-regulated in endomyocardial biopsies of AS patient with or without severe fibrosis. qPCR experiments validated the downregulation of miR-122 and miR-18b in patients with severe fibrosis compared with those with no severe fibrosis and control subjects. These two miRNAs potentially target TGF-β1.

### 3.2. Mitral Valve Disease

Very few studies investigated miRNA profiles in mitral valves from mitral valve disease patients, and the only available studies were designed to assess the influence of atrial fibrillation (AF) more than the effect of the disease itself as compared to controls. Because a recent review has extensively addressed the role of miRNAs in AF [34], this particular topic will not be documented here.

#### 3.2.1. Atrium

The expression of 28 miRNAs was differentially altered in the right atrium of patients with mitral stenosis (MS) and AF as compared to patients with mitral stenosis but no history of AF [35]. Another study showed miRNA dysregulation in MS with AF, with differences between right and left atrium [36]. Finally, miRNA expression profiles were compared in left and right appendages from patients with rheumatic mitral valve disease, and sinus rhythm or AF [37].

#### 3.2.2. Mitral Valves

In a study available in the form of an abstract, high-throughput RNA sequencing coupled with miRNA sequencing was performed to identify novel molecular targets as well as upstream regulators contributing to MMVD [38]. In this study, 67 miRNAs were differentially expressed between normal and myxomatous mitral valves. mRNA levels of the genes encoding TGF-β-induced factor homeobox 1, salt-inducible kinase 1, tissue inhibitor of metalloproteinase 4, and cyclin-dependent kinase inhibitor 1C mRNA levels were decreased in myxomatous tissue, and miRNAs predicted to target these genes (e.g., miR-656, miR-379-3p, miR-664a-3p, and miR-34c-5p) were significantly increased.

A recent study compared miRNA profiles in explanted mitral valves in MMVD and in fibroelastic deficiency (FED), two common types of degenerative mitral valve disease [39]. miR-500, -3174, -17, -1193, -646, -1273e, -4298, -203, -505, and -939 were differentially expressed in MMVP and FED. In silico prediction of potential target genes revealed genes involved in extracellular matrix homeostasis and genes encoding components of mitral valves (e.g., decorin, aggrecan, fibromodulin, α actin 2, extracellular matrix protein 2, desmin, endothelial cell specific molecule 1, and platelet/endothelial cell adhesion molecule 1). The expression of these gene mRNAs was inversely correlated with miRNA expression in patient groups. This study provides first molecular evidence for the existence of distinct mechanisms underlying MMVP and FED.

### 3.3. Circulating miRNA

Specific signatures of circulating miRNAs have been assigned to both acute and chronic CVD, such as AF, CAD, myocardial infarction, heart failure, vascular disease, and cardiac death [40,41]. Cellular source of miRNAs can be diverse, including cardiomyocytes, endothelial cells, platelets and fibroblasts, and release can occur in a disease-specific manner [42]. Whether the release of miRNAs into the circulation is a passive or an active process remains unknown. Circulating miRNAs can be delivered to recipient cells and modify their protein expression pattern [43].

The levels of miRNAs in serum and plasma are reproducible, consistent among individuals. Circulating miRNAs are more stable than mRNAs and seem to be protected from endogenous ribonuclease activity. Circulating miRNAs would indeed be stabilized by the formation of Ago2-miRNA complex and/or protected from degradation by encapsulation in exosomes [44].

Seven studies compared the levels of candidate circulating miRNAs in AS cases vs. controls, with the aim to identify AS specific miRNA (Table 2). Seven miRNAs were described to be differentially expressed in the plasma of AS patients (miR-21 [31], miR-133 [30], miR-1 [32], miR-378 [45], miR-210 and miR-22 [46], and miR-122 [33]). In these studies, high expression of miR-21 correlated with mean transvalvular gradient and LV fibrosis, and miR-378 levels correlated with LV mass index. miR-1 was associated with LV hypertrophy (LVH) and correlated with levels of soluble heart-type fatty acid-binding protein-3 (FABP3), a lipid-binding protein and main target of peroxisome proliferator-activated receptor γ (PPARγ). miR-378 was an indepedent predictor of LVH, while miR-133a predicted LVH reversibility one year after surgery. Preoperative levels of circulating miR-133a were significantly higher in the cohort of AS patients who normalized LV mass after pressure overload release compared with those who maintained residual hypertrophy [30]. Increased miR-210 levels in AS were comparable to increments of BNPs. Furthermore, miR-210 levels were associated with higher mortality after 3.5 year follow-up. Another study compared patients with hypertrophic non-obstructive or obstructive cardiomyopathy with AS patients and described specific upregulation of miR-29c in AS [47]. Recently, Coffey et al. performed whole miRNome profiling in an effort to translate one or more miRNAs into biomarkers for use in calcific AS [48]. Given the established link between obstructive CAD and altered circulating miRNA profile [49], patients with or without CAD were analyzed separately. Circulating whole miRNome profiles discriminate, albeit incompletely, between participants with AS and those without. Two upregulated miRNAs, miR-451a and miR-22-3p, and 2 downregulated miRNAs, miR-24-3p and miR-382-5p, remained significantly different after adjusting for age. In a validation cohort composed of patients with moderate or severe AS, in terms of mean aortic valve maximum velocity, mean pressure gradient, and calculated aortic valve area, only miR-22-3p and miR-382-5p had the expected results on qPCR, and these were observed only in patients with CAD. In agreement with Villar et al. [31], miR-21-5p levels were higher in patients with AS without CAD, but showed no difference between groups in those with CAD. miR-21-5p and miR-382-5p levels showed a statistically significant correlation with maximum transvalvular velocity and mean gradient, but not LV mass index.

In these studies, myocardial and plasma levels of miR-1 [32], miR-21 [31] and miR-133a [30] correlated directly, supporting the myocardium as a relevant source of these miRNA. Notably, changes in valvular miRNA never correlated with modification of their plasma levels. We can therefore anticipate that circulating miRNA will reflect mainly VHD-associated myocardial remodeling and altered function. However, it seems that AS would still be characterized by distinct circulating miRNA profiles. Indeed, Roncarati et al. [50] identified a profile of circulating miRNA that distinguishes patients with hypertrophic cardiomyopathy (HCM) from healthy individuals. Three significantly up-regulated miRNAs, miR-27a, -29a, and -199a-5, correlated with LV mass, whereas only miR-29a correlated with fibrosis. Nevertheless, this profile was different from that of hypertrophy caused by aortic stenosis, and may thus be disease specific. In severe AS, miR-29a levels were not increased relative to the control group.

Circulating miRNAs have not been investigated in human mitral valve diseases. Two studies have been performed in dogs with MMVD. Hulanicka et al. [51] analyzed the expression of nine candidate miRNAs in the plasma of Dachshunds with MMVD and identified two significantly downregulated miRNAs: cfa-miR-30b in dogs with moderate cardiac enlargement and cfa-miR-133b in dogs with congestive heart failure. A second study investigated the expression of 277 miRNA in serum [52]. Dogs with MMVD and mild to moderate cardiac enlargement or with congestive heart failure had four upregulated miRNAs, cfa-miR-103, cfa-miR-98, cfa-let-7b, and cfa-let-7c, while seven others were downregulated (cfa-miR-302d, cfa-miR-380, cfa-miR-874, cfa-miR-582, cfa-miR-490, cfa-miR-329b, and cfa-miR-487b), compared to normal dogs. Expression of six of these miRNAs also significantly differed between dogs with congestive heart failure and those with mild to moderate cardiac enlargement (cfa-miR-582, cfa-miR-487b, cfa-miR-103, cfa-miR-98, cfa-let-7b, and cfa-let-7c)*.* The expression changes were greater as disease severity increased.

## 4. Roles of miRNAs in VHD: Functional Studies

**Figure 1:**
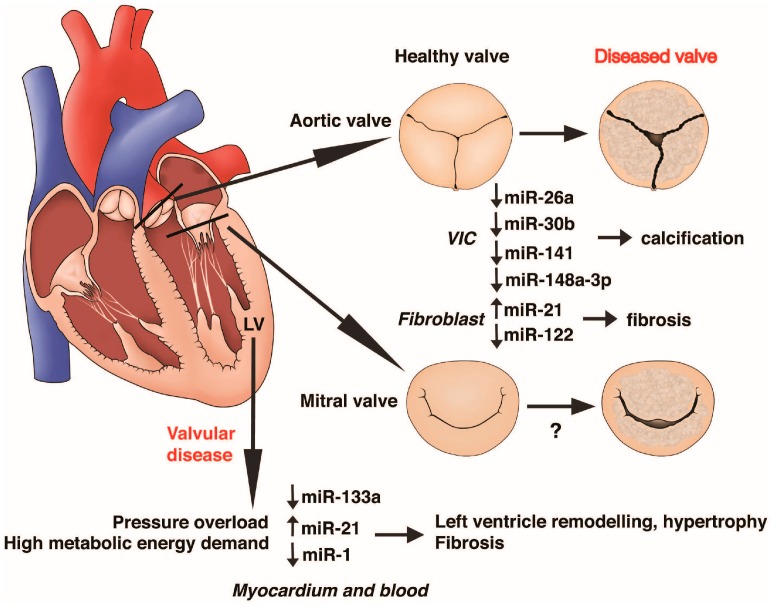
Overview of miRNA function in the development of valvular heart disease. Data are from functional studies on aortic stenosis. Dysregulated miRNA expression has been described in valvular interstitial cells (VIC), fibroblasts, and myocardium. Changes in myocardial and plasma levels of miR-1, miR-21, and miR-133a are correlated. The role of miRNA in human mitral valve diseases remains largely unknown. Up and down arrows depict up- and down-regulation of miRNA expression, respectively.

Few studies have undertaken functional analyses in cellular, mouse or zebrafish models in order to gain mechanistic insight into the role of identified miRNA in aortic stenosis pathophysiology (Table 3, Figure 1). Putative or proven target genes were highlighted.

In a mouse model of pressure overload-induced LV remodelling by transverse aortic constriction, elevated levels of miR-21 were observed, attributing a role for this miRNA in myofibroblast differentiation and maladaptive LV fibrosis by targeting effectors of TGF-β1 signaling, i.e., programmed cell death 4 (PDCD4) and Reversion Inducing Cysteine Rich Protein With Kazal Motifs (RECK), a negative regulator of MMP-9 [31]. Interestingly, the recent study by Garcia et al. [53] indicated that TGF-β1-induced phosphorylation of SMAD2/3 caused the interaction of p-SMAD2/3 with the cytoplasmic endonuclease RNase III DICER1, which promoted pre-miR-21 processing to mature miR-21. In a zebrafish model, miR-21 appeared as a central component of a flow-controlled mechanotransduction system during valvulogenesis acting as a positive regulator of cell proliferation [54], a process that could also be implicated in pathological remodelling of the valves. Thus, there is strong evidence that pressure overload leads to increased myocardial miR-21 in both animal and human models, which controls LV remodelling and fibrosis by acting on myocardial cell differentiation and/or proliferation.

In a general manner, miRNAs have been involved in fibrosis, mainly targeting ECM structural proteins or enzymes involved in ECM remodelling, in pro-fibrotic TGF-β signaling pathways, and connective tissue growth factor (CTGF). They also affect epithelial-to-mesenchymal transition, induce myofibroblast proliferation, and their resistance to apoptosis [55]. More particularly, miR-21 has been involved in fibrosis in the heart, but also in lung and kidney. In different cell types isolated from failing hearts, miR-21 was predominantly upregulated in cardiac fibroblasts, and mediated protection from apoptosis, possibly through activation of extracellular signal-regulated kinase/mitogen activated protein kinase (ERK/MAPK) signaling [56]. Sprouty1, a negative regulator of ERK/MAPK, was identified as the direct target of miR-21. However, in the mice, miR-21 seems not to be essential for ERK/MAPK activation in stress-dependent cardiac remodeling [57]. Further investigations are warranted to determine whether these mechanisms contribute to VHD-associated valvular and LV fibrosis.

Data from AS patients LV biopsies suggest that the miR-133a-Wolf-Hirschhorn Syndrome Candidate 2 (WHSC2) axis could also play a role in the regulation of cardiac hypertrophy [30]. It has been described that miR-133 plays a key role in the control of the trophic state of the heart under normal conditions and that, when miR-133 is down-regulated, such as in the pressure overload condition, the transcriptional derepression of genes encoding proteins that regulate cardiac structure likely contributes to the adverse remodeling response [58,59]. The *WHSC2* gene, also known as *NELF-A*, encodes a component of the Negative Elongation Factor complex involved in the regulation of RNA polymerase II transcription elongation. However, the mechanistic link of the miR-133a-WHSC2 axis with AS pathophysiology remains to be determined.

Pressure overload has also been associated with decreased expression of myocardial miR-1 in a mouse model of transverse aortic constriction and in AS patients [32]. In this study, decreased miR-1 expression correlated directly with increased levels of the cardiac-type fatty acid binding protein 3 (FABP3). Of interest, FABP3 is involved in the control of lipid metabolism through its role in the uptake, intracellular metabolism and transport of long-chain fatty acids. Thus, downregulation of miR-1 in AS may reflect the switch of the myocardium to a high metabolic energy demand, accompanying the cardiac hypertrophy response through FABP3 upregulation and subsequent myocardial metabolic remodeling.

Of note, miR-133 originates from the same bicistronic transcript as miR-1, and it has been reported that both miR-133 and miR-1 play important roles in generating cardiomyocytes from embryonic stem cells and in reprogramming human fibroblasts to cardiac-like myocytes [60,61]. There are only few miRNA families regulating biological processes in the heart, and miR-1 is probably one of the most abundant [62]. miR-1 is upregulated in individuals with coronary artery disease and its overexpression worsens arrhythmogenesis. However, the majority of the studies agree on the fact that miR-1 plays a protective role against cardiac hypertrophy or heart failure by targeting several genes related to hypertrophy, e.g., calmodulin, Mef2a, insulin-like growth factor-1, insulin-like growth factor-1R, and sodium–calcium exchanger. Whether these mechanisms contribute to AS-associated LV hypertrophy remains to be determined.

In the valves, miRNAs may play an important role in osteoblast transition of VICs [63]. Four studies used human aortic VICs and showed functional implication of miR-30b in the prevention of abnormal osteogenesis and apoptosis by direct targeting of Runt-related transcription factor 2 (RUNX-2), SMAD-1, and caspase-3 [24,26]. Likewise, miR-26a represses several calcification-related genes [24], and therefore, downregulation of these miRNAs would alleviate protective mechanisms, leading to valve calcification. Exposure of aortic VICs to cyclic stretch downregulates miR-148-3p, resulting in the activation of inflammation through NF-κB signaling pathway [27]. Thus, abnormal haemodynamics encountered in AS would drive an inflammatory response yielding immune cell recruitment to the valve and subsequent calcification. Using TGF-β-stimulated porcine AVICs, miR-141 was identified to inhibit osteoblastic transition, possibly by repressing BMP-2 [25]. Downregulation of miR-125b in infiltrating macrophages would result in increased levels of the CCL4 chemokine, promoting immune cell recruitment [28]. In fibroblasts, downregulation of miR-122 would contribute to fibrosis, possibly through TGF-β1 upregulation [33].

Among other miRNAs that are downregulated in aortic stenosis, it has been described that miR-210 contributes to osteoblast differentiation by repressing the activity of the TGF-β type I receptor as part of the osteoblast lineage commitment program [64]. Likewise, miR-29 regulates osteoblast differentiation by inhibiting osteonectin expression through the canonical Wnt pathway. However, functional evidence is lacking to determine whether these regulatory mechanisms would apply to valve calcification processes.

Circulating miRNAs could be involved in paracrine communication between blood cells, VEC and VIC, contributing to tissue homeostasis. Changes in haemodynamics may have an effect on miRNA expression that could be responsible for disease progression [65].

In this context, it has been reported that platelet-derived microparticles mediate transfer of their cargo between platelets and other cells [66,67], and disease-associated dysregulation of miRNA content in platelets might therefore affect other cells, serving as paracrine signaling molecules. Several studies on human platelets indicate that miRNA content varies in different disease states [68]. For instance, levels of platelet miRNAs in the circulation have been associated with atrial fibrillation and peripheral vascular disease [69]. Microparticles produced upon platelet activation can be cargos of Ago2-microRNA complexes that are delivered to endothelial cells [70]. Since platelet activation has been associated with calcific AS, platelet-derived miRNAs might thus have important roles as biomarkers of disease diagnosis, prognosis, or treatment. Besides, recent evidence attributes mechanistic roles for platelet-derived miRNAs in haemostasis, thrombosis, and unstable coronary syndromes. Further investigations are required to determine if platelet-derived miRNAs could contribute to VHD progression and serve as biomarkers.

Furthermore, recent evidence indicate that extracellular vesicles derived from smooth muscle cells, VICs and macrophages could mediate calcification in diseased heart valves [71]. These calcifying vesicles are indeed loaded with miRNAs that target genes involved in osteogenic differentiation, among which miR-30 and miR-125b could be particularly relevant to VHD. However, the regulation of EV release, and the mechanisms of interaction between EVs and the extracellular matrix leading to the formation of microcalcifications remain unclear.

Thus, despite the identification of few miRNAs with potential diagnostic or pronostic significance in VHD, most of the studies are still preliminary, because of limited numbers of miRNAs measured or small size of patient cohorts. As a consequence, only modest correlation with disease severity was reported, and identified miRNAs are unlikely to provide sufficient discrimination between heterogeneous groups of patients.

## 5. miRNA Therapeutics

Optimal therapeutic strategy would target early stages of disease development before valve calcification becomes irreversible. Studies indicate that miRNA could be important regulators of both fibrosis and calcific nodule formation. Beyond their potential use as biomarkers of disease onset and progression, miRNA-based therapeutics could also be envisioned.

A single miRNA can target several mRNAs and a group of miRNAs can target genes from the same pathway, suggesting that miRNA targeting could simultaneously affect a number of relevant dysregulated gene networks.

A new class of drugs that specifically targets miRNA is presently in development. Three main approaches have been exploited: expression vectors (miRNA sponges), small-molecule inhibitors and antisense oligonucleotides (ASOs) [72].

It has been proposed that anti-miR-mediated silencing could be a powerful strategy for the treatment of human disease [72]. However, a number of concerns remain before such therapies can be translated to clinical applications. The first difficulty is to find out strategies that would deliver miRNA to target tissues. The major obstacle is the off-target effects. Specificity can be improved by using several miRNAs that target a single mRNA. Increase of desired effect could also be achieved by selecting a miRNA that targets multiple genes within a same pathway.

In aortic stenosis, we can anticipate that anti-miR-21 approaches could be useful to limit cardiac valve or LV fibrosis. miR-133-based therapies may be directed to manipulate the trophic state of the heart. miR-1 or miR-133a mimics would synergize with conventional medical and surgical measures to achieve reverse remodeling of the overloaded LV. Local delivery of mimics of miR-26a, miR-30b, miR-141, or miR-148a-3p could be envisionned to counteract valve calcification.

## 6. Conclusions

Current knowledge allows us to assert that miRNAs might represent important regulators of VHD development. Most studies focused on aortic stenosis, while the role of miRNA in mitral valve diseases remains largely unknown. Notwithstanding, available data suggest that distinct miRNA are dysregulated in aortic and mitral valve diseases, supporting different underlying pathophysiological mechanisms.

In AS, miRNAs could contribute to changes in VIC phenotype that occur under disturbed flow, and they could regulate key processes underlying VHD, i.e., fibrosis, calcification, LV remodelling, thrombosis and inflammation.

These miRNAs certainly warrant further investigation. Future studies using larger patient cohorts as well as animal models will have to be designed to address two important challenges. The first challenge would be to identify miRNA biomarkers consistently dysregulated in specific VHD and at different stages of disease progression, miRNAs that could be markers of patient response to therapy, or that could serve to monitor post-surgery events. We believe that miRNA could be included in a multi-biomarker approach combined with high resolution imaging modalities to assess small changes in the leaflets. Such an approach could provide the potential to obtain a global view on molecular and cellular processes that have an impact on disease onset, and could be the basis for a more personalised and predictive medicine. The second challenge would focus on the assessment of miRNA function in order to evaluate the possibility to target miRNA and related proteins as new therapeutic avenues.

## Figures and Tables

**Table 1:** miRNA profiling studies for valvular heart disease (VHD).

Origin	Methods	Dysregulated miRNAs	Study Details
*Aortic stenosis*			
Aortic valve leaflets [24]	Microarray qPCR	Downregulated: 26a, 30b, 195	9 patients with BAV (AS vs. aortic insufficiency requiring AVR)
Aortic valve leaflets [26]	qPCR	Downregulated: 30b	10 AS patients requiring AVR (calcific vs. adjacent tissue)
Aortic valve leaflets [27]	qPCR	Downregulated: 148a-3p	4 BAVs vs. healthy aortic valves
Aortic valve leaflets [28]	Microarray qPCR	Upregulated: 125b Downregulated: 374b*, 602, 939	20 calcific AS valves vs. 6 control valves
Aortic valve leaflets [25]	Microarray qPCR	Upregulated: 151-3p, 152, 030e, 032, 145, 768-5p, 190, 373* Downregulated: 141, 370, 022, 1972, 330-5p, 566-pre, 1469, 1908, 648-pre, 637, 1282, 622, 486-3p, 187, 1909*, 564, 027a, 194*, 125b-1-pre, 551a-pre, 575, 585-pre, 211-pre, 449b*, 124-3-pre, 185-pre, 1202	19 BAVs vs. 17 TAVs from AS patients requiring AVR
LV intraoperative biopsies [29]	qPCR	Upregulated: 133a	46 AS patients requiring AVR
LV intraoperative biopsies [31]	qPCR	Upregulated: 21	75 AS patients requiring AVR vs. 32 surgical controls
LV intraoperative biopsies [32]	qPCR	Downregulated: 1	5 AS patients before TAVI vs. healthy controls
Endomyocardial biopsies [33]	Microarray qPCR	Downregulated: 18b, 122	28 AS patients with severe myocardial fibrosis vs. non-severe fibrosis
LV intraoperative biopsies [30]	qPCR	Upregulated: 133a	74 AS patients requiring AVR
*Mitral valve disease*			
Tissues from the right and the left atrial appendages [37]	Microarray qPCR	Upregulated: 4484 Downregulated: 1, 23b-3p, 26a-5p, 30c-5p, 125b-5p, 133b, 143-3p, 145-5p, 4454	18 rheumatic mitral valve disease patients (10 AF vs. 8 SR) requiring mitral valve surgery
Tissues from the left atrial appendage [36]	Microarray qPCR	Upregulated: 466, 574-3p, 3613-3p Downregulated: 1, 26a-5p	12 patients with mitral stenosis (6 SR, 6 AF) requiring mitral valve surgery
Mitral valve leaflets [38]	RNA-seq	Upregulated: 656, 379-3p, 664a-3p, 34c-3p	10 patients with myxomatous mitral valve vs. 10 controls
Mitral valve leaflets [39]	whole genome miRNA qPCR	Upregulated: 500, 3174 Downregulated: 17, 203, 505, 646, 939, 1193, 1273e, 4298	10 myxomatous mitral valve prolapse vs. fibroelastic deficiency

Abbreviations: AF, atrial fibrillation; AS, aortic stenosis; AVR, aortic valve replacement; BAV, bicuspid aortic valve; LV, left ventricle; TAV, tricuspid aortic valve; TAVI, transcatheter aortic valve implantation; qPCR, quantitative real-time PCR.

**Table 2:** Studies of circulating miRNAs as potential biomarkers for aortic stenosis.

Patients	Methods	Findings
75 AS patients requiring AVR vs. 32 surgical controls [31]	qPCR	High expression of miR-21 correlates with mean transvalvular gradient and LV fibrosis
5 AS patients before TAVI vs. healthy controls [32]	qPCR	Decreased miR-1 correlates with increased soluble FABP3 in AS patients upon LVH
112 patients with moderate to severe AS vs. 40 healthy controls [45]	qPCR	Lower levels of miR-1, miR-133a, and miR-378 in AS patients miR-378 levels correlate with LV mass index Independent predictor of LVH in AS
57 patients with moderate to severe AS vs. 10 healthy controls [46]	qPCR	Increased miR-210 levels in AS patients comparable to increment in NT-proBNP levels miR-210 levels associate with higher mortality (3.5 year follow-up)
74 AS patients requiring AVR [30]	qPCR	miR-133a as a positive predictor of the hypertrophy reversibility after surgery
94 severe AS patients (with or without CAD) vs. 101 controls [48]	Microarray qPCR	miR-22-3p is upregulated in AS, while miR-382-5p was downregulated, only in patients with CAD miR-21-5p levels is higher only in AS without CAD Only miR-21-5p and miR-382-5p levels correlate weakly with measures of disease severity
23 patients with HNCM, 28 HOCM, 47 AS, 22 healthy controls [47]	qPCR	miR-29c is specifically upregulated in aortic stenosis

Abbreviations: AS, aortic stenosis; AVR, aortic valve replacement; CAD, coronary artery disease; HNCM, hypertrophic non-obstructive cardiomyopathy; HOCM: hypertrophic obstructive cardiomyopathy; LVH, left ventricular hypertrophy; TAVI, transcatheter aortic valve implantation; LV, left ventricle.

**Table 3:** miRNAs related to fibrocalcific valve remodelling or LV hypertrophy.

miRNA	Model	Target Genes	Findings
*Aortic valvular interstitial cells*			
miR-30b	Human AVICs	*RUNX2*, *SMAD1*, *CASP3*, *SMAD3*, *BMP2*, *NOTCH1*	miR-30b prevents osteogenesis and apoptosis through direct targeting of Runx2, Smad1, and caspase-3 [26] Downregulates calcification-related gene pathways [24]
miR-26a	Human AVICs	*ALPL*, *BMP2*, *SMAD1*	miR-26a represses several calcification-related genes and increases mRNA level of genes that may have roles in inhibiting calcification (*JAG2*, *SMAD7*) [24]
miR-148a-3p	Human AVICs	*IKBKB*	Exposition of AVICs to cyclic stretch represses miR-148a-3p, which activates the NF-κB-dependent inflammatory signalling pathway [27]
miR-141	Porcine AVICs	*BMP2*	miR-141 inhibits osteoblastic transition of TGF-β stimulated VIC, possibly by targeting BMP-2 [25]
*Fibroblasts*			
miR-122	Human fibroblasts	*TGFB1*	Down-regulation of miR-122 contributes to fibrosis, possibly through TGF-β1 up-regulation [33]
miR-21	NIH3T3 fibroblast cell line	-	Stimulation with TGF-β induces p-SMAD2/3 interaction with DICER1, which promotes pre-miR-21 processing to mature miR-21 [53]
*Left ventricle*			
miR-1	LV biopsies from AS patients	*FABP3*	myocardial miR-1 expression was decreased whereas the circulating sFABP3 level was increased in AS patients compared with healthy subjects miR-1 modulates the cellular and secreted levels of FABP3, which may represent an indirect biomarker in the plasma, reflecting the cellular activity of miR-1 [32]
miR-21	LV biopsies from AS patients	-	LV myocardium from AS patients exhibits overexpression of DICER1 mRNA that is directly related to the expression of TGF-β1, and its effectors SMAD2, and SMAD3, and to pre-miR-21 [53]
miR-21	LV biopsies from AS patients	*RECK*, *PDCD4*	miR-21, its targets, and effectors of TGF-β signaling predict the variance of myocardial collagen [31] miR-21 plays a role in myocardial fibrosis
miR-133a	LV biopsies from AS patients	*WHSC2*	Combined myocardial miR-133a and clinical parameters predict LV mass normilisation 1 year after AVR [29,30]
*Immune cells*			
miR-125b	THP-1 monocytic cells	*CCL4*	Chemokines are among the most upregulated genes in calcific AS valves, and a downregulation of miR-125b in infiltrating macrophages leads to increased CCL4 levels [28]
*Mouse model of tranverse aortic constriction*			
miR-1	Myocardium of transgenic mice overexpressing cardiac miR-1	*FABP3*	Ventricule pressure overload leads to cardiac hypertrophy, and results in a switch of the myocardium to a high metabolic energy demand, which is paralleled by an increase of IGF-1 levels, and decreased miR-1 Inverse relationship between myocardial expression of miR-1 and circulating levels of FABP3 [32]
miR-21	Mouse myocardium	-	TGF-β induces miR-21 up-regulation in myocardium under pressure overload, which contributes to maladaptive remodelling and fibrosis [31]
miR-21	Mouse myocardium	-	Pressure overload upregulates myocardial DICER DICER transcript levels correlate directly with TGF-β1, SMAD2, and SMAD3 [53]
*Zebrafish development*			
miR-21	Zebrafish heart valve	*spry2*, *pdcd4a*, *ptenb*	miR-21 is a central component of a flow-controlled mechanotransduction system during heart valve formation, acting as a positive regulator of cell proliferation [54]

Abbreviations: AS, aortic stenosis; AVIC, aortic valve interstitial cells; AVR, aortic valve replacement; BMP-2, Bone morphogenetic protein-2; CCL4, Chemokine (C–C motif) ligand 4; DICER1, Double-Stranded RNA-Specific Endoribonuclease; LV, left ventricle; SMAD2/3, SMAD Family Member 2/3; p-SMAD2/3, phosphorylated-SMAD2/3; TGF-β, transforming growth factor-β.

## Abbreviations

AF	Atrial fibrillation
AS	Aortic stenosis
AVIC	Aortic valve interstitial cells
AVR	Aortic valve replacement
BAV	Bicuspid aortic valve
BMP-2	Bone morphogenetic protein-2
BNP	B-type natriuretic peptide
CAD	Coronary artery disease
CCL4	Chemokine (C–C motif) ligand 4
HNCM	Hypertrophic non-obstructive cardiomyopathy
HOCM	Hypertrophic obstructive cardiomyopathy
LV	Left ventricle
LVH	Left ventricular hypertrophy
MMVD	Myxomatous mitral valve degeneration
SMAD2/3	Mothers against decapentaplegic homolog (SMAD) family member 2/3
p-SMAD2/3	Phosphorylated-SMAD2/3
TAC	Transverse aortic constriction
TAV	Tricuspid aortic valve
TAVI	Transcatheter aortic valve implantation
TGF-β	Transforming growth factor-β
VEC	Valve endothelial cells
